# Supplementary material for: Extrusion Foaming of Lightweight Polystyrene Composite Foams with Controllable Cellular Structure for Sound Absorption Application
Source: Polymers (Basel). 2019 Jan 9;11(1):106. doi: 10.3390/polym11010106 (PMC6401839; doi:10.3390/polym11010106)
Supplement: Supplementary file 1 [file polymers-11-00106-s001.pdf]

Supplementary Information

Extrusion Foaming of Lightweight Polystyrene Composite Foams with Controllable Cellular Structure for Sound Absorption Application

Yanpei Fei[a], Wei Fang[a], Mingqiang Zhong[a,], Jiangming Jin[b,]*, Pin Fan[a], Jingtao Yang[a], Zhengdong Fei[a], Lixin Xu[a,]*, Feng Chen[a,]*

[a] *College of Materials Science and Engineering, Zhejiang University of Technology, Hangzhou 310014, P R China.*

[b] *College of Mechanical Engineering, Zhejiang University of Technology, Hangzhou 310014, P R China.*

*Corresponding authors: Jiangmin Jin, Email: jjm@zjut.edu.cn; Lixin Xu, Email: gcsxlx@zjut.edu.cn; Feng Chen, Email: chenf@zjut.edu.cn

1. The characterization of PS/50%Lignin

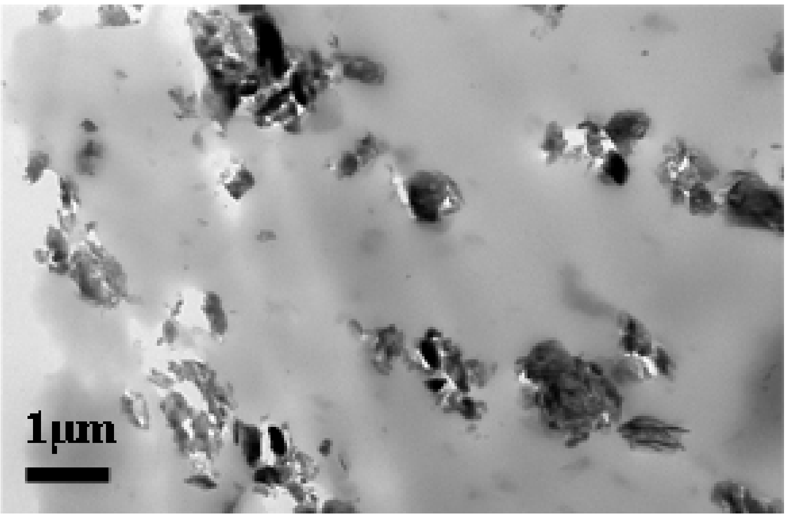

Fig. S1 The TEM image of PS/Lignin composite with 50 wt% lignin content.

2. The density of PS composite foams

Table S1 The density of PS composite foams.

Sample	ρ (g/cm^3)	Sample	ρ (g/cm^3)
PS	0.09	PS/0.2%MWCNT	0.08
PS/10%Lignin	0.08	PS/0.5%MWCNT	0.09
PS/20%Lignin	0.07	PS/1%MWCNT	0.11
PS/30%Lignin	0.08	PS/0.2%mGr	0.08
PS/40%Lignin	0.10	PS/0.5% mGr	0.09
PS/50%Lignin	0.33	PS/1% mGr	0.10
